# Estimation of ribosome profiling performance and reproducibility at various levels of resolution

**DOI:** 10.1186/s13062-016-0127-4

**Published:** 2016-05-10

**Authors:** Alon Diament, Tamir Tuller

**Affiliations:** Biomedical Engineering Department, Tel Aviv University, Tel Aviv-Yafo, Israel; The Sagol School of Neuroscience, Tel Aviv University, Tel Aviv-Yafo, Israel

**Keywords:** Ribosome profiling, mRNA translation, Next generation sequencing

## Abstract

**Background:**

Ribosome profiling (or Ribo-seq) is currently the most popular methodology for studying translation; it has been employed in recent years to decipher various fundamental gene expression regulation aspects.

The main promise of the approach is its ability to detect ribosome densities over an entire transcriptome in high resolution of single codons. Indeed, dozens of ribo-seq studies have included results related to local ribosome densities in different parts of the transcript; nevertheless, the performance of Ribo-seq has yet to be quantitatively evaluated and reported in a large-scale multi-organismal and multi-protocol study of currently available datasets.

**Results:**

Here we provide the first objective evaluation of Ribo-seq at the resolution of a single nucleotide(s) using clear, interpretable measures, based on the analysis of 15 experiments, 6 organisms, and a total of 612, 961 transcripts. Our major conclusion is that the ability to infer signals of ribosomal densities at nucleotide scale is considerably lower than previously thought, as signals at this level are not reproduced well in experimental replicates. In addition, we provide various quantitative measures that connect the expected error rate with Ribo-seq analysis resolution.

**Conclusions:**

The analysis of Ribo-seq data at the resolution of codons and nucleotides provides a challenging task, calls for task-specific statistical methods and further protocol improvements. We believe that our results are important for every researcher studying translation and specifically for researchers analyzing data generated by the Ribo-seq approach.

**Reviewers:**

This article was reviewed by Dmitrij Frishman, Eugene Koonin and Frank Eisenhaber.

**Electronic supplementary material:**

The online version of this article (doi:10.1186/s13062-016-0127-4) contains supplementary material, which is available to authorized users.

## Background

Translation has a major role in the regulation of gene expression and significantly affects various fundamental intracellular processes and biomedical phenomena [1–7]. It is an energetically most costly process, and each of its initiation, elongation and termination steps is tightly regulated [8, 9]. The most prominent experimental technique for studying translation in recent years has been ribosome profiling (RP; or Ribo-seq) [10]. This approach enables high-throughput monitoring of ribosomal density along genes by utilizing deep sequencing methods and has been employed to decipher fundamental gene expression regulation aspects in recent years [10–16].

Ribosome profiling is based on deep-sequencing of ribosome protected mRNA fragments from living cells, such that the sequence of each fragment indicates the position of a translating ribosome on the transcript [10]. The experiment comprises of the following main steps: preparation of the biological samples; sample lysis; nuclease footprinting, in which mRNA that is not protected by ribosomes is digested; ribosome (monosome) recovery; linker ligation; rRNA depletion; library sequencing, followed by bioinformatics analysis of the sequences [17]. Various variants of the experimental protocol have been developed, and many steps in the protocol need to be optimized according to the relevant organism and experimental system [18]. Specifically, it has been shown that the choice methods for translation inhibition [19, 20], RNA digestion enzyme and concentration [17, 18], monosome purification [18] and rRNA depletion [18, 20] all affect the quality of the resultant data. Moreover, several methods have been applied for mapping the sequenced ribosome protected fragments, and specifically the location of the A-site (or P-site) of the ribosome, to the genome [10, 17, 18, 21–23].

It has been suggested, by utilizing various methods as well as RP, that the speed by which ribosomes progress along the mRNA is affected by different local features of the coding sequence [24, 25]. However, despite its promising throughput, analysis of RP data has led to contradictory conclusions between studies, such as the heating the debate around the determinants of ribosome elongation speed. These include, among others, the following issues: wobble base-pairing was suggested to slow elongation down in *C. elegans* and human [26], in agreement with previous (non-RP) experiments [27, 28], but no evidence for this was found in recent studies that analyzed *S. cerevisiae* profiles [21, 29]. Positively-charged amino acids were shown to slow elongation down in multiple organisms [25, 30], in agreement with previous experiments [31], but no evidence for this was found in a recent study [21]. The local secondary structure of the mRNA was shown to have a relation between its folding energy and elongation rate [25, 32, 33], in agreement with previous reports [34], but no evidence for this was found in other studies [21, 30]. Finally, the effect of optimal/non-optimal codons on elongation rate and the relation between the latter and tRNA abundance has been reported [11, 35] and denied [21, 30, 36–38], while being verified by other experimental means [39–42].

While the consistency and reproducibility of RP estimation over *entire* coding regions was provided in the first paper about this method [10], no similar analysis has been provided for RP estimations in *local* regions of the coding region, *and particularly not in a large-scale approach encompassing multiple datasets in various organisms and based on various conventional protocols.* Thus, the performance of the RP method has yet to be accurately/objectively and thoroughly evaluated. The aim of the current study is to provide for the first time such an objective evaluation which should be robust to the different RP analyses approaches and simple to interpret. In addition, we discuss how our analysis can be used as a tool in future studies of local translation aspects via RP.

To this end, we analyze multiple RP datasets containing experimental replicates in order to determine the consistency and reproducibility of the profiles in closely related repetitions. We show that in most of the studied experiments to date, the level of reproducibility in measured ribosomal densities at nucleotide (or a few nucleotides) scale is considerably lower than previously thought, and argue that some of the aforementioned contradictions may be attributed to the resolution and relatively high ‘noise’ levels in RP data when studying ribosome densities in short fragments of the coding regions. We believe that our results are important for every researcher studying translation and specifically for researchers analyzing data generated by the RP approach.

## Results

### The robustness of local RP measurements is usually more than one order of magnitude lower than global RP measurements

**Fig. 1:**
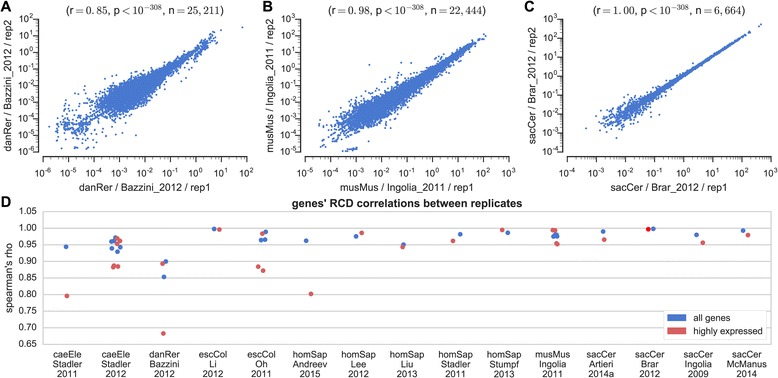
Comparison of ribosomal densities. **a** Scatter plot for all genes in zebrafish, where the x-axis represents the Ribo-seq read count density (RCD) of a gene in one replicate of the Bazzini-2012 dataset [14] (WT, 6hpf), while the y-axis represents RCD in a second replicate. Spearman’s rho, *p*-value and the number of points are denoted above the plot. This is the lowest correlation obtained between replicates in this analysis. **b** Same for the Ingolia-2011 dataset [38] (w/LIF, 60s CHX). This is the median correlation obtained between replicates in this analysis. **c** Same for the Brar-2012 dataset [12] (meiotic stage). This is the highest correlation obtained between replicates in this analysis. **d** The correlation between all pairs of replicates for all genes and for the subset of 20 % highly expressed genes in each dataset

**Fig. 2:**
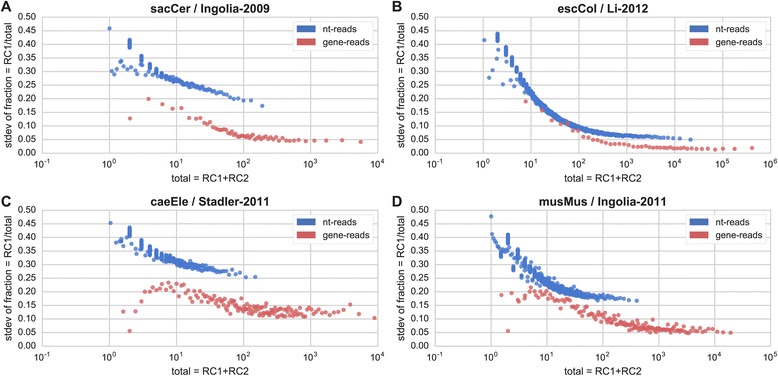
Local and global reproducibility in RP replicates. The figure presents the inter-replicate variance for a measured nucleotide position in the transcript (blue) and for complete genes (red). Y-axis is the standard deviation of the fraction of total read counts (RCs) measured in replicate 1 (read count 1, RC1), while the X-axis denotes the total number of read counts in that position in both replicates (RC1, RC2). Each point (bin) is based on the standard deviation of 1000 positions in the dataset for nt-reads, or 100 positions for gene-reads. The confidence in the measurement increases (the variance decreases) with the total read count, as expected. The difference between the two profiles indicates that additional noise and bias exist at the nucleotide level, that is considerably higher than in the gene level. This noise/difference is evident even after the profiles reach plateau, and its gain varies from experiment to experiment. Repeated for: **a** Ingolia-2009 [10]; **b** Li-2012 [36]; **c** Stadler-2011 [26]; **d** Ingolia-2011 [38]

**Fig. 3:**
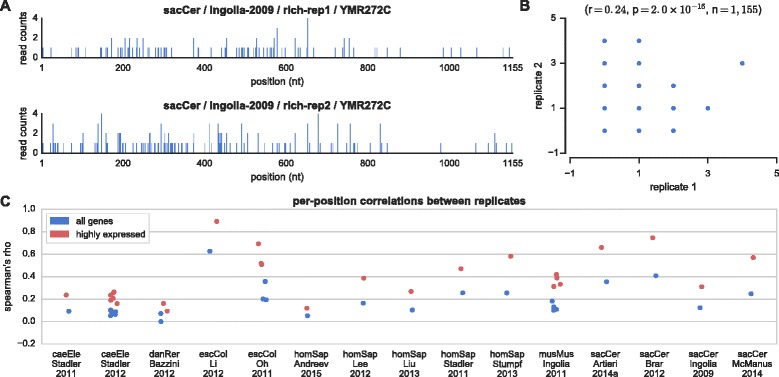
Comparison of position-specific occupancies. **a** Two measured profiles for the *S. cerevisiae* gene YMR272C from replicates in the Ingolia-2009 dataset [10]. Bars represent the approximated location of the A-site (15 nt downstream from the 5’ end of the measured read). The average coverage in this profile is 8.4 % (7.4 % in the first replicate and 9.5 % in the second one). This is the 3^rd^ quartile transcript according to coverage in this dataset (its coverage is higher than that of 75 % of the genes). **b** Scatter plot of the respective read counts in all nucleotide positions, in each of the replicates for the transcript in panel (**a**). Spearman’s rho, p-value and the number of points are denoted above the plot. **c** The median per-position correlation between all pairs of replicates for all genes and for the subset of 20 % highly expressed genes in each dataset

Correlations between experimental replicates in the ribosome profiling literature are often reported to be very high [10, 23, 43], similar in level to RNA-seq measurements [10] (Fig. 1). We analyzed 15 ribosome profiling experiments containing multiple replicates from 6 organisms and confirmed that, indeed, the correlations between the Ribo-seq read count densities (RCD) of genes in different experimental replicates are high in most cases (r between 0.85 and 1.00). However, while representing every gene with a single value is informative enough for certain types of analyses, many of the questions that ribosome profiling was designed to answer require reproducibility at a much-higher resolution, up to the nucleotide level. It should be noted that local RP measurements (e.g., nucleotide positions) are subject to additional biases and noise that are not as dominant at the global, gene level. For example, one source for such type of noise could be related to inefficient halting of elongation that at some probability allows for additional cycles of elongation to occur [39]. Thus, previous analyses of replicate consistency at the global level cannot predict reproducibility at the local level (Fig. 2). We therefore tested for the first time the reproducibility of ribosome occupancy profiles at the nucleotide level (Fig. 3). The coverage (percentage of nucleotides in the transcript to which at least one ribosomal footprint mapped) of most transcripts in the genome is low, leading to sparse profiles with many differences between repetitions. For example, a typical gene in terms of coverage in the Ingolia-2009 [10] dataset appears in Fig. 3a, with a coverage as low as 8 % (this is in fact the 3^rd^ quartile, with a coverage higher than that of 75 % of the genes). The correlation between measured read counts at every nucleotide position in replicates for this transcript was 0.24 (*p* = 2x10^−16^) (Fig. 3b), a significant but rather weak correlation (only 5.8 % of the variance of the read count profile of one replicate can be explained by the second one). We computed per-position correlations for the entire transcriptome between replicates in the 15 experiments (Fig. 3c). For example, the median correlation between two transcripts appearing in the Ingolia-2009 dataset [10] is 0.12 (*p* = 5.7x10^−8^). Similarly, in most ribosome profiling experiments analyzed we found that the median correlation in the genome was below 0.4 (16 % of the variance of the read count profile of one replicate can be explained by the second one), indicating that the profiles are not reproducible at the nucleotide level. The 20 % highly expressed genes in each experiment showed higher correlations, but still typically below 0.6 (36 % of the variance of the read count profile of one replicate can be explained by the second one). Highly expressed genes have a higher RCD and tend to have profiles of higher coverage, leading to a higher number of reads per position and to a higher confidence in their count per position, which promotes reproducibility (Fig. 3c). It should be noted that we obtained similar results for datasets that were generated using various RP protocol variants, including such that avoided pre-treatment of the samples with cycloheximide before lysis [23, 26, 36, 44]. Similar conclusions regarding the local and global reproducibility of RP were obtained via different measures, demonstrating the robustness of these conclusions (Fig. 2).

### Estimation of the increase in local RP robustness as a function of the level of resolution

**Fig. 4:**
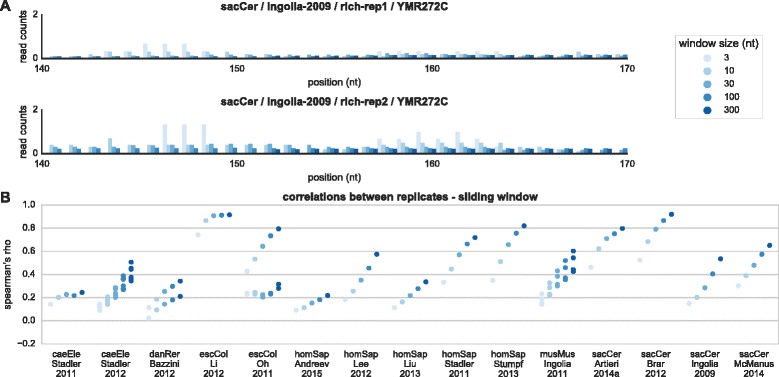
Smoothing of profiles using sliding windows. **a** Zoom-in on YMR272C (see also Fig. 3a), showing the smoothed profile for various averaging windows. The correlation between the profiles increases with the window size. **b** The median per-position correlation between all pairs of replicates after smoothing with 5 different sliding windows

**Fig. 5:**
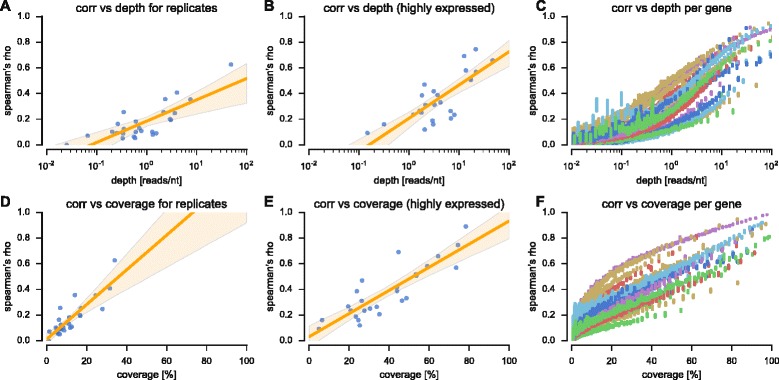
Expected reproducibility given experiment depth and coverage. **a** Per-position correlations between profiles (median per experimental replicate pair) against the combined replicates’ depth (reads per nucleotide position). Regression line (orange) shows the linear regression between correlation and log-depth, with the 95 % confidence intervals of the model parameters marked within the orange area. **b** Same for the subset of 20 % highly expressed genes in each dataset. **c** Here correlations and depth were computed for individual genes and binned into 100 sets of genes with similar depth. Each line denotes the mean of the bin and the 95 % confidence interval around the mean. The 26 replicate pairs were independently colored and their genes show consistent behavior according to similar model parameters (replicates from the same experiment/organism may have similar shades). **d** Same as (**a**) for correlation versus coverage. **e** Same as (**b**) for correlation versus coverage. **f** Same as (**c**) for correlation versus coverage

In order to estimate the resolution of profiles better, and to test whether the integration of additional reads can improve correlations, we utilized sliding window averaging to smooth the profiles (Fig. 4a). The smoothed profiles showed increasing per-position correlations for growing sliding window sizes, with the maximal correlation obtained for the largest window size (300 nt), as expected from undersampled profiles (the median correlation was 0.15 for a 3 nt-window, 0.23 for a 10 nt-window, 0.29 for a 30-nt window and 0.45 for a 300 nt-window, see Fig. 4b). The smoothed profiles integrate over more reads than the raw profiles in order to estimate the occupancy at a given position, interpolate values for missing positions, and are less sensitive to small shifts in the mapping of reads. We tested to what extent the coverage and depth (average count of reads mapped to each position in the transcriptome, i.e., the total read count density) of an experiment can predict the reproducibility of the results. To this end, we plotted the median per-position correlation of all pairs of replicates against the depth of the combined replicates (details in Methods), for all genes (Fig. 5a), and for the subset of highly expressed genes (Fig. 5b). The results suggest that sequencing depth should be exponentially increased to raise the correlation between profiles (a correlation of 0 for 0.02 reads/nt in Bazzini-2012 [14] up to a correlation of 0.63 for 48.7 reads/nt in Li-2012 [36]); thus our analysis provides a way to estimate the expected intra coding sequence reproducibility when deciding on the sequencing depth. Similar results were obtained when plotting the correlation against the depth of individual genes (Fig. 5c). In addition, we plotted the same per-position correlations against the average coverage of replicates (Fig. 5d–e), with results suggesting a linear relation between coverage and the expected correlation (an increase of 10 % in coverage is related to an increase of 0.09 in the correlation coefficient). When looking at the correlations and coverage of individual genes, many of the experiments show a linear relation, with some small deviations (Fig. 5f). While each experiment shows a trend that is consistent with a single linear model, the model parameters differ between experiments. This diversity may be attributed to other parameters that determine the amount of noise in the experiment, such as the protocol being used, the conditions during its execution, the organism studied etc.

### Typically 30 % of the RP extreme peaks are reproducible

**Fig. 6:**
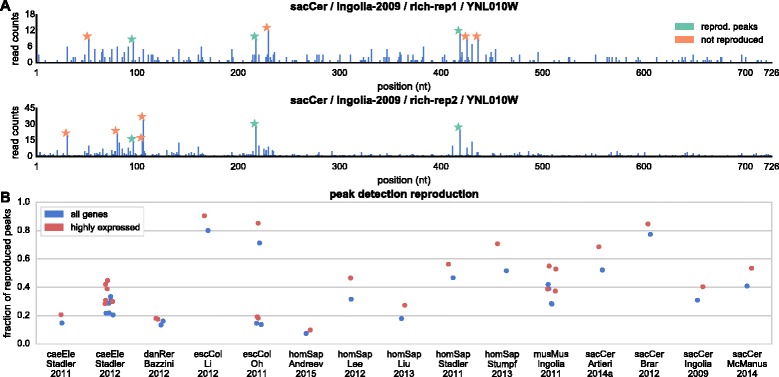
Peak detection consistency. **a** Two measured profiles for the *S. cerevisiae* gene YNL010W from replicates in the Ingolia-2009 dataset [10]. Bars represent the approximated location of the A-site (15 nt downstream from the 5’ end of the measured read). Detected peaks in each profile are denoted with a star. 43 % of all identified peaks have corresponding peaks within 3 nt of their identified position in the other replicate. This is the 4^th^ quintile transcript according to its peak detection reproducibility score in the dataset. **b** Peak detection score between all pairs of replicates, for all genes and for the subset of 20 % highly expressed genes in each dataset

In the next step, we tested the reproducibility of extreme values in the profiles. Peaks in ribosome profiles have been suggested to represent pauses in translation and have been analyzed to determine pausing factors in the sequence [13, 36, 38, 45]. Peaks vary in their frequency between experiments and are typically detected in 0.1–1 % of the genome (details in Methods). We defined a peak detection reproducibility score as the fraction of total detected peaks in two profiles (replicates) that have corresponding peaks in the other replicate, within an error of 3 nt (Fig. 6a). We computed this score for all genes in all pairs of replicates, and found that median peak reproducibility over all experiments is 30 % (Fig. 6b). As with the previous tests, highly expressed genes showed higher consistency (the median peak reproducibility is 40 %). These results demonstrate that also *extreme* peaks tend to be irreproducible.

## Discussion

It is important to mention that we limited our analyses only to a number of aspects that may affect reproducibility. The variance between the studied datasets suggests that *many* other factors play a significant role in determining the consistency between replicates and the conclusions of different studies. Among others, additional noise and biases may rise from various further sources: from steps in the experimental protocol such as elongation halting [19, 46], RNA digestion, rRNA filtering, etc.; from genome construction and annotations; from read mapping biases; from analysis of a (very) small subset of reliable genes. Thus, as the analysis of the datasets was performed here in a unified manner (where methods usually vary between studies) and focused on replicates from the same experiment (conducted in very similar conditions), *the results reported here are only an upper bound on reproducibility of Ribo-seq analysis results, which is expected to be much lower in practice* (specifically when comparing the results obtained based on different experimental protocols and computational procedures).

Our study demonstrates that usually we should be very cautious when analyzing RP at the intra-coding region nucleotide(s) level; if such an analysis is performed it should be based on statistical approaches tailored for dealing with this challenging data or should include various filtering steps. We also suggest to evaluate the expected reproducibility before starting the analysis/experiments, as described here.

Indeed, more elaborate models can be utilized to deal with bias and noise in the data without discarding information. Ingolia et al. [38] improved the mapping of the A/P-sites by estimating the location of the site along reads that mapped directly upstream the start/sop codons. Oh et al. [23] assigned ribosome protected footprints in 1–16 nt long smoothed footprints, depending on the footprint length, thus adjusting the effective resolution of the profiles. Artieri and Fraser [21] performed bias correction by normalizing the observed RP read counts using the corresponding RNA-seq read counts at the same positions. Recently, a multi-scale approach for analyzing RP profiles at an adaptive resolution while correcting for biases has been proposed by Gritsenko et al. [47]. In Dana and Tuller [11] the noise in RP read counts was modeled as a combination of independent random variables (signal and noise), in order to filter out the latter.

One possible approach to alleviate the issues discussed here is to conduct larger/high-coverage experiments, as we show that reproducibility is strongly correlated with depth and coverage. Sequencing depth can also be partially increased by improved preparation of the RP library in order to avoid contamination, e.g., by rRNA fragments [18]. However, it should be noted that the plots in Fig. 5 are in logarithmic scale, and the reproducibility does not grow very quickly. For instance, in order to achieve an expected correlation of 0.9 between replicates, according to Fig. 5b, we would need a sequencing depth of 105 reads per base. Such a transcriptome-wide sequencing depth would require approximately 400 M mappable reads for a small transcriptome like *E. coli*’s, but closer to 4,000 M mappable reads for the human, mouse or zebrafish transcriptomes – 2-3 orders of magnitude higher than recently published RP papers. Authors should be encouraged to report the extent and scale of their experiments clearly in every study; this is specifically important when local nucleotide-level signals are reported. Another approach that is more readily available is rigorous statistical handling of the data. The experience gained since ribosome profiling was first proposed has led to the development of a number of techniques to reduce noise in the data. The most common of which is gene filtering, either according to read count threshold [10, 14, 23, 36, 38, 43, 48, 49], coverage threshold [11, 13], or by comparing to a reference null distribution [50]. Reads are usually filtered according to their length, with approaches that vary from strict [30, 37] to more relaxed ones [10]. Acceptable alignments to the genome are also subject to constraints, from 0 mismatches and unique alignment [21, 37], to 2 mismatches and handling of multiple alignments [11]. Another form of filtering is ignoring the 5’-end and/or 3’-end of ORF [11, 13, 21]. When detecting transcripts with differential changes in read-counts, genes with inconsistent results between replicates can be filtered out [43].

Here we provide an additional approach for handling data as the plots reported here can be used for evaluation of the RP data and for choosing the resolution of the analyses according to the desired reproducibility level.

The challenges in analyzing RP data that arise from this report call for the continuation of development and enhancement of robust and tailored statistical methods.

## Conclusions

In this study we provide, for the first time, an objective evaluation of RP reproducibility at different levels of intra-coding region resolution for various organisms and RP protocols.

Our main conclusions are that that the level of noise in measured ribosomal densities at nucleotide(s) scale is considerably higher than previously thought, as signals at this level are not reproduced well in experimental replicates. Our analyses indicate that this holds even when ignoring 80 % of the genes with lower expression levels in the genome. Furthermore, various protocol variants, including such that avoided pre-treatment of the samples with cycloheximide before lysis, showed similar levels of performance in our analyses. This issue has important implications to many of the intra-coding region analyses done on ribosome profiling data, and may explain some of the discrepancies between the conclusions of different studies in the field; nevertheless, it hasn’t been systematically studied and discussed in the literature.

## Methods

### Genome sequences

Transcript sequences were obtained from EnsEMBL [51]: *S. cerevisiae* (R64-1-1, release 78, 12/2014), *M. musculus* (GRCm38, release 78, 12/2014), *H. sapiens* (GRCh38, release 80, 5/2015), *D. rerio* (GRCz10, release 81, 7/2015), *C. elegans* (WBcel235, release 81, 7/2015), *E. coli* (K-12 MG1655 release 121, accessed 28/07/15). We used annotated UTRs where available, and otherwise used up to 100 nt upstream and downstream the ORF that didn’t overlap another ORF. Each gene was represented by its longest annotated transcript.

### Mapping reads

We selected a wide range of datasets from multiple studies, labs, protocol variants and organisms that contained at least two replicates that could be analyzed and compared. Details on datasets and replicates appear in Table 1. We trimmed adaptors from the reads using Cutadapt [52] (version 1.8.3), and utilized Bowtie [53] (version 1.1.1) to map them to the transcriptome (representing each gene by its longest annotated transcript). In the first phase, we discarded reads that mapped to rRNA and tRNA sequences with Bowtie parameters ‘–n 2 –seedlen 23 –k 1 --norc’. In the second phase, we mapped the remaining reads to the transcriptome with Bowtie parameters ‘–v 2 –a --strata --best --norc –m 200’. When the 3’ adaptor contained polyA we tried to extend alignments to their maximal length by comparing the polyA with the aligned transcript until reaching the maximal allowed error (2 mismatches across the read, with 3’-end mismatches avoided). We filtered out reads longer than 34 nt and shorter than 27 nt. Unique alignments were first assigned to the ribosome occupancy profiles. For multiple alignments, the best alignments in terms of number of mismatches were kept. Then, multiple aligned reads were distributed between locations according to the distribution of unique ribosomal reads in the respective surrounding regions. To this end, a 100 nt window was used to compute the read count density RCD_*i*_ (total read counts in the window divided by length, based on unique reads) in vicinity of the *M* multiple aligned positions in the transcriptome, and the fraction of a read assigned to each position was RCD_*i*_/∑_*j* = 1_^*M*^RCD_*j*_. The location of the A-site was approximated by a 15 nt shift from the 5’ end of the aligned read [21, 26, 35]. We verified that our mapping approach yields similar profiles to previously published ones [14, 49] (Additional file 1). While additional (or less) heuristics can be applied during mapping, our mapping approach serves as a baseline to compare the replicates using a unified method, thus eliminating differences that often arise from the choice of mapping and/or analysis methods between studies. Optimizing the mapping procedure of Ribo-seq data remains an open question and deferred to future studies.

**Table 1:** Dataset summary

Organism	Dataset	Condition	Treatment	Replicate	Type	Accession
*C. elegans*	Stadler_2011 [26]	L1	post CHX	rep1 rep2	biological	SRR405089 SRR405091-2
	Stadler_2012 [44]	L1	post CHX	rep1 rep2 rep3 rep4	biological	SRR522871 SRR522872 SRR522896 SRR522897
*D. rerio*	Bazzini_2012 [14]	WT, 2hpf WT, 6hpf	pre/post CHX	rep1 rep2 rep1 rep2	biological	SRR392998-9 SRR393000-1 SRR393006-7 SRR393008-9
*E. coli*	Li_2012 [36]	MOPS	post GMPPNP+ Chloramphenicol	rep1 rep2	biological	SRR407274-5 SRR407276-7
	Oh_2011 [23]	DSP	pre/post Chloramphenicol rapid filtration	rep1 rep2 rep3	biological	SRR364364 SRR364366 SRR364368
*H. sapiens*	Stadler_2011 [26]	HeLa, CHX	post CHX	rep1 rep2	technical	SRR407637 SRR407638
	Lee_2012 [54]	HEK293T, CHX	pre/post CHX	rep1 rep2	technical	SRR618770 SRR618771
	Liu_2013 [45]	HeLa-tTA, K71M	pre/post CHX	rep1 rep2	biological	SRR619099 SRR619100
	Stumpf_2013 [50]	HeLa, G1	pre/post CHX	rep1 rep2	biological	SRR970490 SRR970538
	Andreev_2015 [48]	HEK293T, control	post CHX	rep1 rep2	biological	SRR1173905 SRR1173909-10
*M. musculus*	Inoglia_2011 [38]	mESC, noLIF-36 h mESC, yesLIF	pre/post CHX	rep1 rep2 rep1 rep2 rep3	biological	SRR315620-2 SRR315623 SRR315601-2 SRR315624-6 SRR315627
*S. cerevisiae*	Ingolia_2009 [10]	YPD	pre/post CHX	rep1 rep2	biological	SRR014374-6 SRR014377-81
	Brar_2012 [12]	meiotic	pre/post CHX	rep1 rep2	biological	SRR387904 SRR387905
	Artieri_2014 [43]	YPD, mixed \w *S. paradoxus*	pre/post CHX	rep1 rep2	biological	SRR1040415 SRR1040423, SRR1040427
	McManus_2014 [49]	YPD	pre/post CHX	rep1 rep2	biological	SRR948553 SRR948555

Details for all analyzed datasets are provided. The Treatment column denotes which drug was used to arrest translation and whether it was added pre- and/or post-lysis

### Replicate testing

Data analysis was performed in Python 3.4 (Anaconda distribution, version 2.3.0) and plotting was done using the Seaborn package (version 0.7.0). All tests in this study are based on comparing a pair of replicates. To this end, we generated all unique pairs between experimental replicates (a total of 26 pairs from 15 publications/datasets). Some of the analyses, such as coverage and depth calculation, were performed independently for each replicate and then averaged or summed to assign the pair with a single value (for example, see Fig. 5a, and details below). When taking the subset of highly expressed genes, we analyzed genes that were in the top 20 % of genes’ ribosomal densities in both replicates. All analyses were performed only on ORFs.

### Correlations

All correlations are Spearman rank correlations unless stated otherwise. Ribo-seq read count densities (RCD) were computed by summing all reads that mapped to the ORF and dividing by ORF length (see Fig. 1a-c). Per-position correlations were computed separately for each gene by computing the correlation between two replicate profiles, including all positions in the ORF. The median correlation of all genes in the genome was used as a summary statistic in Figs. 3c and 4b.

### Profile smoothing

Smoothing was done using a sliding window in various sizes. Each “nucleotide” in the smoothed profile represents the average over 3, 10, 30, 100 or 300 nucleotides around it in the raw profile (see Fig. 4a). Averaging was calculated uniformly over the window. Genes shorter than the window were discarded.

### Depth and coverage

Depth was defined as the average number of times every nucleotide in the genome appeared in the 5’ of a ribosome protected fragment (read). That is, the read count density of the genome (total read count divided by the total length of ORFs). This value is directly related to the sequencing depth of the experiment. When computed for individual genes (see Fig. 5c), the read count density of the gene (total read count divided by ORF length) was utilized as depth. In order to represent a replicate pair we utilized their total depth, i.e., the sum of their depths.

Coverage was defined as the percentage of non-zero positions in a gene, and the total coverage was defined as the average coverage of all genes. For a replicate pair, the coverage was the average coverage of the two. This value is not only related to sequencing depth, but also to the number of unique ribosome protected fragments that were sampled in the library (which is related to the number of cells, number of mRNA molecules and number of ribosomes on each molecule).

### Peak detection score

We defined a peak detection threshold that was calculated for each gene independently. The threshold was set to be 3 standard deviations above the median, as calculated over all non-zero positions in the gene. When testing for peak detection reproducibility we accepted the reproduction of a peak if the other replicate had a detected peak within 3 nt upstream or downstream the original peak. The peak detection score is the probability of a detected peak to be reproduced, as estimated by the fraction of all identified peaks in the transcriptome that were successfully reproduced in the two replicates (see Fig. 6a).

## Reviewers’ comments

### First Review

#### Reviewer’s report 1: Dmitrij Frishman, Technische Universität München, Germany

**Reviewer summary**

This is a very useful and timely study, which might explain, at least to some extent, the recent controversial results in analyzing various aspects of mRNA structure, function, and evolution based on ribosomal profiling data. The paper is very well written and its technical quality is very good.

**Reviewer recommendations to authors**

- What I found a little confusing is the statement on page 7, which seems to suggests that reproducibility of the results quickly grows with increased sequencing depth. What are the implications of this finding? Does that mean that the problem can be fixed by deeper sequencing?
- The authors implemented their own pipeline for processing NGS data and obtaining ribosomal occupancy profiles from each experiment. I would be interested to know whether the profiles they derived are similar to those provided by the authors of the original experimental studies. This could provide some insight as to how much depends on the particular approach for processing reads.
- Would it make sense to present results separately for technical and biological replicates (Table 1)?

**Minor issues**

- Why is there only the red point in Fig. 1d for the dataset “sacCerBrar2012” ?
- X-axis label in Fig. 2 is confusing and not explained (RC1,RC2)
- Explain the meaning of the yellow area in Figs. 5a, b, d, e

Authors’ response: *Thank you for the valuable comments. Below are our point-by-point responses.*

- *The reproducibility of the results indeed grows with the sequencing depth/percentage of sequence covered by reads. However, it should be noted that the plots in Fig.*5*are in logarithmic scale, and the reproducibility does not grow very quickly. For instance, in order to achieve an expected correlation of 0.9 between replicates, according to Fig.*5*b, we would need a sequencing depth of 105 reads per base. Such a transcriptome-wide sequencing depth would require approximately 400 M mappable reads for a small transcriptome like E. coli’s, but closer to 4000 M mappable reads for the human, mouse or zebrafish transcriptomes – 2-3 orders of magnitude higher than recently published papers. Finally, there are many additional sources of error/bias in RP experiments, as discussed in the manuscript.*
- *We included in the revised manuscript a comparison between the profiles we generated and two previously published profiles in S. cerevisiae and in D. rerio (see Additional file*1*). The results show a high correlation between the two mappings in both cases. However, it should be noted that in most cases aligned/further-processed profiles were not provided by the authors. In addition, even if such profiles exist, they were often generated using different reference genomes/gene annotations as these are frequently updated. The comparison is further complicated when additional non-trivial steps were taken to produce the profiles, such as smoothing or various methods for the estimation of the location of the A-site of the ribosome.*
- *Provided that only two of the replicates are technical replicates, we leave it to the reader.*
- *We fixed Fig.*1*D where one red dot covered a blue dot with a similar y-axis value.*
- *We added a clearer description to the legend of Fig.*2*.*
- *The area denotes the 95 % confidence interval of the regression parameters. We added a clarification to the figure legend.*

#### Reviewer’s report 2: Eugene Koonin, National Institutes of Health, United States

**Reviewer summary:** In this straightforward paper, Diament and Tuller analyze the consistency between experimental replicates in ribosomal profiling experiments and show that it is high at the level of whole genes but low at the level of individual nucleotides or short segments. Thus, at present the RP data appear not to be truly informative for the interpretation of the role of local features (such as, for instance, short hairpins in mRNA) which could explain various contradictions that have accumulated in the literature. Quite strikingly, the local accuracy is shown to be low even for subsets of highly expressed genes. As far as I can see, the analysis is well done and carefully presented. The authors make several suggestions how to extract more information from RP results without discarding the data or seeking a major experimental breakthrough. I believe these findings are important for any researcher involved in RP experiments or using the RP data for other analysis, which is a large and growing segment of the scientific community.

**Reviewer recommendations to authors:** I think all is well done, no suggestions.

**Minor issues:** No such issues.

Authors’ response: *We thank Prof. Koonin for his endorsement.*

#### Reviewer’s report 3: Frank Eisenhaber, Agency for Science, Technology and Research Singapore

**Reviewer summary:** The authors review the ribosome profiling (ribo-seq) methodology as a tool for studying translation and the biological results obtained with it as reported in recent literature.

**Reviewer recommendations to authors**

1. The article is written as if all readers are well informed about the ribo-seq method and its possible applications. I suggest the authors to add another section at the beginning of the results where they describe the procedure in detail including the post-experimental data processing and conclusion chain (instead of just referring to the original articles. Along this description, the authors can critically remark where are issues of complications with regard to experimental or numerical inaccuracies, assumptions that are not fully supported by evidence, etc. In the later part of the MS, these issues can then be argued with the help of data taken from the 15 studies used.
2. What is labelled “conclusions” in the MS, is rather an elongated discussion section.

**Minor issues:** none.

Authors’ response: *Thank you for your comments. Below are our point-by-point responses.*

1. *We added a description of the ribo-seq method to the introduction of the paper, along with references to recent papers that review the experimental protocol in detail and point to sensitive steps in the process.*
2. *We re-organized the manuscript and divided the last section into discussion and conclusions.*

### **Second Review**

#### Reviewer’s report 1: Dmitrij Frishman, Technische Universität München, Germany

I am happy with the revision.

#### Reviewer’s report 2: Eugene Koonin, National Institutes of Health, United States

No comments

#### Reviewer’s report 3: Frank Eisenhaber, Agency for Science, Technology and Research Singapore

It appears to me that Dima Frishman has been labelled as reviewer two times in the answers. I guess that my name should appear as referee 3.

Authors’ response: *Sorry. This was fixed.*

## Additional file

Additional file 1: Comparison of mapped RP profiles in this study with previously published ones. **(A)** Scatter plot for all yeast genes, where the x-axis represents the RPKM of a gene in the profiles generated in this study from a replicate of the McManus-2014 dataset (GSM1259974), while the y-axis represents the RPKM of a gene in the profiles published by the authors as bedGraph files in sacCer3 strand-specific genomic coordinates. Since the bedGraph profiles were smoothed by the authors by assigning values to all bases covered by the aligned ribosome protected fragment, we performed similar smoothing to our profiles using a 30 nt window. Spearman’s rho, p-value and the number of points are denoted above the plot. **(B)** Histogram of the position-specific correlations for yeast genes between the mapped profiles in this study and the ones provided by McManus et al. (median correlation *r* = 0.90). **(C)** Same as (A), for the Bazzini-2012 dataset based on smoothed profiles provided by the authors in GSM854439 in zv9 genomic coordinates (not strand-specific). **(D)** Same as (C), for the Bazzini-2012 dataset (median correlation *r* = 0.75). (PNG 839 kb)
