# Supplementary figures and images for: Estimation of ribosome profiling performance and reproducibility at various levels of resolution
Source: Biol Direct. 2016 May 10;11:24. doi: 10.1186/s13062-016-0127-4 (PMC4862193; doi:10.1186/s13062-016-0127-4)

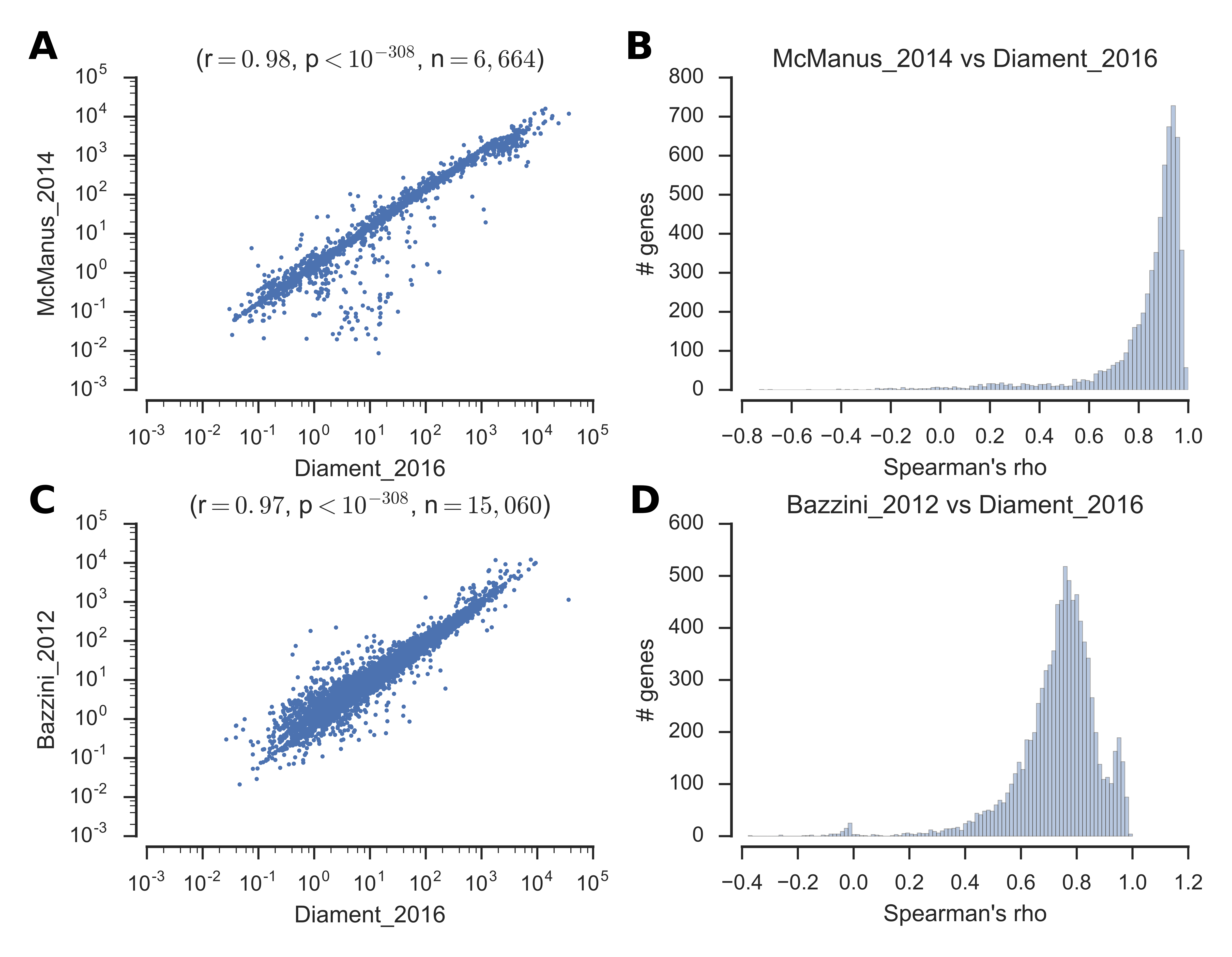

Supplement: Additional file 1: — Comparison of mapped RP profiles in this study with previously published ones. (A) Scatter plot for all yeast genes, where the x-axis represents the RPKM of a gene in the profiles generated in this study from a replicate of the McManus-2014 dataset (GSM1259974), while the y-axis represents the RPKM of a gene in the profiles published by the authors as bedGraph files in sacCer3 strand-specific genomic coordinates. Since the bedGraph profiles were smoothed by the authors by assigning values to all bases covered by the aligned ribosome protected fragment, we performed similar smoothing to our profiles using a 30 nt window. Spearman’s rho, p-value and the number of points are denoted above the plot. (B) Histogram of the position-specific correlations for yeast genes between the mapped profiles in this study and the ones provided by McManus et al. (median correlation r = 0.90). (C) Same as (A), for the Bazzini-2012 dataset based on smoothed profiles provided by the authors in GSM854439 in zv9 genomic coordinates (not strand-specific). (D) Same as (C), for the Bazzini-2012 dataset (median correlation r = 0.75). (PNG 839 kb) [file 13062_2016_127_MOESM1_ESM.png]
